# Mechanisms of curcumin-induced gastroprotection against ethanol-induced gastric mucosal lesions

**DOI:** 10.1007/s00535-017-1385-3

**Published:** 2017-08-30

**Authors:** Renata Czekaj, Jolanta Majka, Katarzyna Magierowska, Zbigniew Sliwowski, Marcin Magierowski, Robert Pajdo, Agata Ptak-Belowska, Marcin Surmiak, Slawomir Kwiecien, Tomasz Brzozowski

**Affiliations:** 1 Neurology Clinic Zeromski Hospital, Cracow, Poland; 2 Department of Physiology, Jagiellonian University Medical College, 16 Grzegorzecka Street, 31-531 Cracow, Poland

**Keywords:** Curcumin, Gastric blood flow, Prostaglandin, Nitric oxide, Afferent sensory fibres, Calcitonin gene related peptide, Transient potential vanilloid receptor, Gastrin

## Abstract

**Background:**

Curcumin, a pleiotropic substance used for centuries in traditional medicine, exhibits antioxidant, anti-inflammatory and antiproliferative efficacy against various tumours, but the role of curcumin in gastroprotection is little studied. We determined the effect of curcumin against gastric haemorrhagic lesions induced by 75% ethanol and alterations in gastric blood flow (GBF) in rats with cyclooxygenase-1 (COX-1) and COX-2 activity inhibited by indomethacin, SC-560 or rofecoxib, inhibited NO-synthase activity, capsaicin denervation and blockade of TRPV1 receptors by capsazepine.

**Methods:**

One hour after ethanol administration, the gastric mucosal lesions were assessed by planimetry, the GBF was examined by H_2_ gas clearance, plasma gastrin was determined by radioimmunoassay, and the gastric mucosal mRNA expression of Cdx-2, HIF-1α, HO-1 and SOD 2 was analysed by RT-PCR.

**Results:**

Curcumin, in a dose-dependent manner, reduced ethanol-induced gastric lesions and significantly increased GBF and plasma gastrin levels. Curcumin-induced protection was completely reversed by indomethacin and SC-560, and significantly attenuated by rofecoxib, L-NNA, capsaicin denervation and capsazepine. Curcumin downregulated Cdx-2 and Hif-1α mRNA expression and upregulated HO-1 and SOD 2, and these effects were reversed by L-NNA and further restored by co-treatment of L-NNA with l-arginine.

**Conclusions:**

Curcumin-induced protection against ethanol damage involves endogenous PG, NO, gastrin and CGRP released from sensory nerves due to activation of the vanilloid TRPV1 receptor. This protective effect can be attributed to the inhibition of HIF-1α and Cdx-2 expression and the activation of HO-1 and SOD 2 expression.

**Electronic supplementary material:**

The online version of this article (doi:10.1007/s00535-017-1385-3) contains supplementary material, which is available to authorized users.

## Introduction

Curcumin is a hydrophobic polyphenol that imparts the yellow colour to the spice obtained from the *Curcuma longa* root [1, 2]. The first descriptions of the use of curcumin in traditional Chinese medicine date back to the Tang Dynasty around 700R AD [3]. In ancient medicine, curcumin was also used to treat gastrointestinal (GI) diseases such as indigestion, flatulence, diarrhoea, and even gastric and duodenal ulcers [4]. Recent studies have proposed anti-carcinogenic activity of curcumin, as this compound has been proven to exert a therapeutic effect on several human cancers including oesophageal, gastric, and small and large intestinal cancers [5–7]. Curcumin has also shown therapeutic potential in the treatment of liver diseases, irritable bowel syndrome (IBS), Crohn’s disease, colitis, and bacterial and parasitic infections of the GI tract [5–8].

Curcumin is an effective scavenger of reactive oxygen and nitrogen metabolites, and exhibits antioxidant and anti-inflammatory activity in the upper and lower GI tract comparable to that exhibited by non-steroidal anti-inflammatory drugs (NSAIDs) [7, 9–11]. Curcumin was found to reduce the expression of mRNA and protein of pro-inflammatory COX-2, iNOS and 5-lipoxygenase (5-LOX) enzymes and suppressed the expression of various proinflammatory cytokines including TNF-α, IL-1, IL-6, IL-8 and interferon gamma (INF-γ), all essential in the mechanism of inflammation and carcinogenesis [12–14].

Despite the proven multi-target, anti-inflammatory properties of curcumin, to date only a few experimental studies have investigated the protective efficacy of curcumin in the stomach against the formation of acute experimental gastric mucosal lesions [15, 16]. As a consequence, little is known about the mediating factors and mechanisms of the potential protective effects of curcumin in the stomach after injury by necrotizing agents such as ethanol.

Therefore, we investigated the mechanisms underlying the gastroprotective effects of curcumin against acute gastric mucosal lesions induced by ethanol, which is known as a strong mucosal damaging agent that causes mucosal injury through its direct contact with the gastric mucosa. In particular, we attempted to determine the role played by the gastric blood flow (GBF), and major gastroprotective factors such as endogenous prostaglandins (PG) and nitric oxide (NO) known to cooperate in the mechanism of gastric mucosal integrity [17, 18], in the mechanism of curcumin-induced gastric protection against ethanol injury using the selective and non-selective COX-1 and COX-2 inhibitors and NO-synthase inhibitor, L-NNA. Previous studies have documented that the capsaicin-sensitive afferent fibres releasing vasodilatory neuropeptides, such as calcitonin gene-related peptide (CGRP), play a central role in gastroprotection [19, 20]. The binding sites for capsaicin, a selective stimulator of these afferent fibres, have been identified and are referred to as TRPV1 [21]. Importantly, TRPV1 is expressed in afferent nerves, and its activation results in the release of vasodilatory neuropeptides, including CGRP [22]. Interestingly, curcumin has the same vanilloid ring pharmacophore as capsaicin, and this vanillyl structure is considered important for curcumin's affinity for and activation of TRPV1 [23, 24]. We have endeavoured to identify the potential contribution of sensory neuropeptides released from sensory afferent nerves such as CGRP and the involvement of TRPV1, as well as PG and NO, in the mechanism of the gastroprotective action of curcumin against ethanol injury. Furthermore, we determined the gastric mucosal expression of pro-inflammatory markers HIF-1α and caudal type homeobox 2 (Cdx-2), both of which are also recognized as tumour markers [25, 26], and the expression of antioxidant enzymes HO-1 and SOD 2 [27, 28] in gastric mucosa exposed to ethanol with or without pretreatment with curcumin.

## Materials and methods

The study was conducted in 168 Wistar rats of both sexes, weighing between 200 and 250 g, which were deprived of food for 24 h before each experiment and placed in individual Bollman-type cages with free access to tap water. The study was approved by the local ethical committee at Jagiellonian University Medical College and conducted in accordance with the Helsinki Declaration.

### Experimental design and treatments

In subsequent studies, five major series (A, B, C, D and E) of experiments (consisting of 6–8 rats each) were carried out. Series A was used to determine the effect of pretreatment with vehicle (saline, i.g.) and curcumin (Sigma-Aldrich, Schnelldorf, Germany), administered exogenously in doses ranging from 2.5 mg/kg i.g. to 100 mg/kg i.g., against the gastric mucosal lesions induced by 75% ethanol. For comparison, pretreatment with a common proton pump inhibitor, omeprazole (20 mg/kg i.g.; Sigma-Aldrich, Schnelldorf, Germany), was employed, and rats pretreated with this proton pump inhibitor received 1 ml of 75% ethanol (i.g.) 30 min later. Subsequent series B, C, D and E were carried out to determine the involvement of PG-COX and NOS-NO systems and capsaicin-sensitive sensory innervation and vanilloid receptors, respectively, in curcumin-induced gastric protection against ethanol-provoked mucosal injury.

In series B, designed to examine the contribution of endogenous PG to the gastroprotective effects of curcumin, several groups of rats, each consisting of 6–8 animals, were pretreated 30 min before the i.g application of 75% ethanol with either (1) vehicle (saline); (2) curcumin (standard dose of 50 mg/kg i.g.) alone; (3) curcumin (50 mg/kg i.g.) administered with or without SC-560 (5 mg/kg i.g. Cayman Chemical, Ann Arbor, USA.), the selective COX-1 inhibitor [29]; (4) curcumin (50 mg/kg i.g.) administered with or without rofecoxib (10 mg/kg i.g. Pfizer, Illertissen, Germany), the highly selective COX-2 inhibitor [29]; or (5) curcumin (50 mg/kg i.g.) administered without or with indomethacin (5 mg/kg i.p.), a non-selective COX-1 and COX-2 inhibitor [30].

In series C, designed to examine the role of NO in curcumin-induced gastroprotection against ethanol damage, rats were pretreated with L-NNA (20 mg/kg i.p., Sigma-Aldrich, Schnelldorf, Germany), the non-selective NOS inhibitor, administered with or without l-arginine (200 mg/kg i.g., Sigma-Aldrich, Schnelldorf, Germany), a substrate for NO-synthase [28, 29], combined with vehicle or curcumin (50 mg/kg i.g.) and followed 30 min later by i.g. application of 1 ml of 75% ethanol.

### Capsaicin-induced functional ablation of sensory nerves and treatment with capsazepine

In a separate series D group of rats, the irreversible functional ablation of visceral-sensory fibres was performed by the subcutaneous (s.c.) application of capsaicin administered in neurotoxic doses according to the method described previously by our group [20]. In this set of experiments, capsaicin (Sigma-Aldrich, Schnelldorf, Germany) was injected s.c. in incremental doses of 25, 50 and 50 mg/kg, respectively, over a 3-day period (a total dose of 125 mg/kg). For each day of capsaicin administration, rats received a small dose of phenobarbital to counteract respiratory impairment resulting from daily dosing of this agent. The effectiveness of denervation was verified by a blink test to confirm the loss of corneal reflex after administering a drop of diluted capsaicin directly into the conjunctival sac [20]. Only rats without corneal reflex confirm sensory denervation, were used to examine the effect of capsaicin denervation on curcumin-induced gastroprotection for at least 2 weeks after administration of the last dose of capsaicin.

In series E rats, the effect of capsazepine (Sigma-Aldrich, Schnelldorf, Germany), an inhibitor of TRPV-1 receptors [20], on curcumin-induced protection against ethanol injury was determined. Rats with intact or capsaicin-ablated sensory nerve fibres, or those pretreated with vehicle (saline i.g.) or capsazepine (5 mg/kg i.g.), received curcumin (50 mg/kg i.g.), with or without CGRP (10 µg/kg s.c., Sigma-Aldrich, Schnelldorf, Germany), and 30 min later vehicle-control and capsazepine-treated rats were exposed to 75% ethanol (1 ml/rat i.g.), administered via orogastric tube as described above.

### Examination of GBF and determination of area of gastric lesions in rats with or without pretreatment with curcumin

For the GBF measurement, the animals were anesthetized with pentobarbital (50 mg/kg i.p.) 1 h after administration of ethanol. After opening the abdominal cavity, the gastric blood flow (GBF) was determined using an H_2_-gas clearance technique as described previously [20, 29, 31]. The results were expressed as a percentage of the flow recorded in intact gastric mucosa. The stomach was removed and dissected along the greater curvature, and macroscopic evaluation of the area of gastric mucosal lesions was performed with a planimeter (Morphomat, Carl Zeiss, Berlin, Germany), as described in detail elsewhere [29, 31].

### Histological evaluation of gastric lesions

Standardized biopsy specimens from the corpus of the stomach, incorporating the total length of the gastric wall, were prepared using 10% buffered formalin for fixation. The samples were embedded in paraffin, followed by5 µm sectioning and staining with hematoxylin and eosin for histology evaluation. A Nikon microscope equipped with a Microplan II digital image system was used for histological examination.

### Determination of plasma gastrin levels in vehicle- and curcumin-pretreated rats subjected to 75% ethanol

Blood samples were taken from selected study groups to determine the concentration of gastrin by radioimmunoassay (RIA) using specific antibodies, as described previously [28, 29]. Briefly, blood samples were collected from vena cava of vehicle-control rats and those pretreated with curcumin, and were placed in tubes containing disodium acetate. The samples were centrifuged for 15 min (4000 rpm), and the blood plasma was stored at −20 °C until RIA analysis. The concentration of gastrin in plasma was determined using anti-gastrin antibodies (rabbit serum 4562, kindly provided by Dr. J. F. Rehfeld, University of Copenhagen, Denmark), with a final dilution of 1:280000 [29]. The antibodies used in our study recognized gastrin-17 and gastrin-34 in equal measure. The effectiveness of gastrin detection, measured as the sensitivity of the method, amounted to 2.5 pmol/l, while the precision of the method ranged from 88 to 92%.

### Determination of mRNA for HIF-1α, Cdx-2, HO-1 and SOD 2 expression in gastric mucosa by RT-PCR

Biopsy specimens of the gastric mucosa were also taken to determine the RT-PCR expression of mRNA for Cdx-2, HIF-1α, HO-1 and SOD 2 in the gastric mucosa collected from intact rats and those who received 75% ethanol, with or without the pretreatment with vehicle (saline) or curcumin applied in a gastroprotective dose of 50 mg/kg i.g. with or without concurrent treatment with L-NNA (20 mg/kg i.g) alone or combined with l-arginine (200 mg/kg i.g.). Samples of the gastric mucosa (about 200 mg) were collected in Eppendorf tubes at 0 °C using laboratory microscope slides and then immediately immersed in liquid nitrogen, and stored at −80 °C until the RNA isolation procedure. The RNA was isolated from the gastric mucosa using the method described by Chomczynski and Sacchi with Trizol (Invitrogen, Carlsbad, CA, USA) according to the manufacturer’s protocol [32]. The cDNA was synthesized from total cellular DNA (5 µg) using the Reverse Transcription System (RTS, Promega, Madison, USA). The PCR reaction was conducted in an automatic DNA thermal cycler using 1 µg cDNA and Promega PCR reagents. To amplify the HIF-1α, Cdx-2, HO-1 and SOD 2 DNA, specific DNA primers were used (Sigma-Aldrich, St. Louis, MO, USA) whose sequences, along with annealing temperature and size of their respective products, are presented in Table S1. In order to verify the integrity of the RNA, a control amplification of β-actin was performed using the same samples (Tab. S1). PCR products were separated by electrophoresis on a 2% agarose gel containing 0.5 µg/ml of ethidium bromide and then visualized when exposed to UV light. The location of the expected PCR products was confirmed by using a control set of PCR products (O’GeneRuler 50 bp DNA). The densitometry method (Gel-Pro Analyser, Fotodyne Incorporated, Hartland, WI, USA) [29] was employed to compare the expression of HIF-1α, Cdx-2, HO-1 and SOD 2 with that of β-actin obtained from the immunoreactive areas of the gastric mucosa from intact rats as well as in those obtained from curcumin-pretreated rats exposed to 75% ethanol.

### Statistical analysis

Results of the experiment were expressed as mean ± SEM and the statistical analysis was performed with ANOVA test and Tukey post hoc test where appropriate. Differences between estimates of effects were considered significant at *p* < 0.05.

## Results

**Fig. 1:**
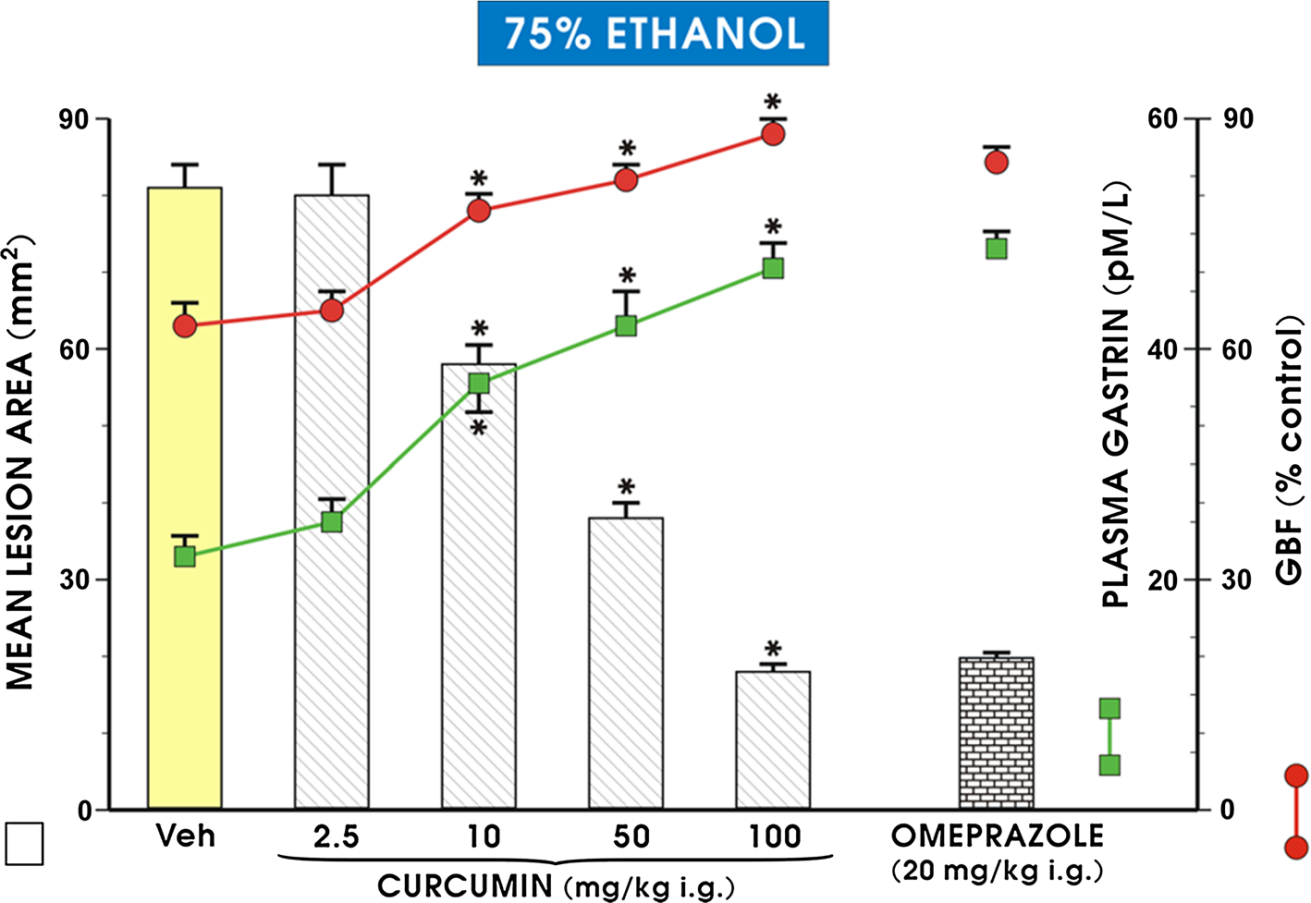
Mean area of ethanol-induced gastric lesions, alterations in gastric blood flow (GBF) and plasma gastrin levels in rats pretreated intragastrically (i.g.) with vehicle (saline) or curcumin applied i.g. in graduated doses from 2.5 mg/kg to 100 mg/kg and, for comparison, with the proton pump inhibitor omeprazole (20 mg/kg i.g). The results are mean ± SEM obtained from seven rats per group. An *asterisk* indicates a significant change (*p* < 0.05) relative to the vehicle-control values

As presented in Fig. 1, intragastric administration of 1 ml of 75% ethanol in vehicle-pretreated rats resulted in the formation of necrotic gastric mucosal lesions, and this effect was accompanied by a significant decrease in the GBF (*p* < 0.05) relative to the respective values of GBF in intact gastric mucosa. The plasma gastrin level in intact rats averaged about 22 pM, and the i.g. application of curcumin at a dose of 2.5 mg/kg had no significant effect on this plasma gastrin level or the area of gastric lesions and GBF relative to vehicle-control animals. Pretreatment with curcumin, applied i.g. in graduated doses of 10, 50 and 100 mg/kg, significantly reduced the area of ethanol-induced gastric lesions, by about 29, 67 and 89% (*p* < 0.05), respectively, and these effects were accompanied by a significant increase in the GBF and plasma gastrin levels relative to the respective values in vehicle-pretreated control rats (*p* < 0.05; Fig. 1). Pretreatment with omeprazole, applied i.g. in a dose of 20 mg/kg, significantly reduced the area of gastric lesions and increased the GBF, similar to that seen with curcumin administered i.g. in a dose of 100 mg/kg (*p* < 0.05).

**Fig. 2:**
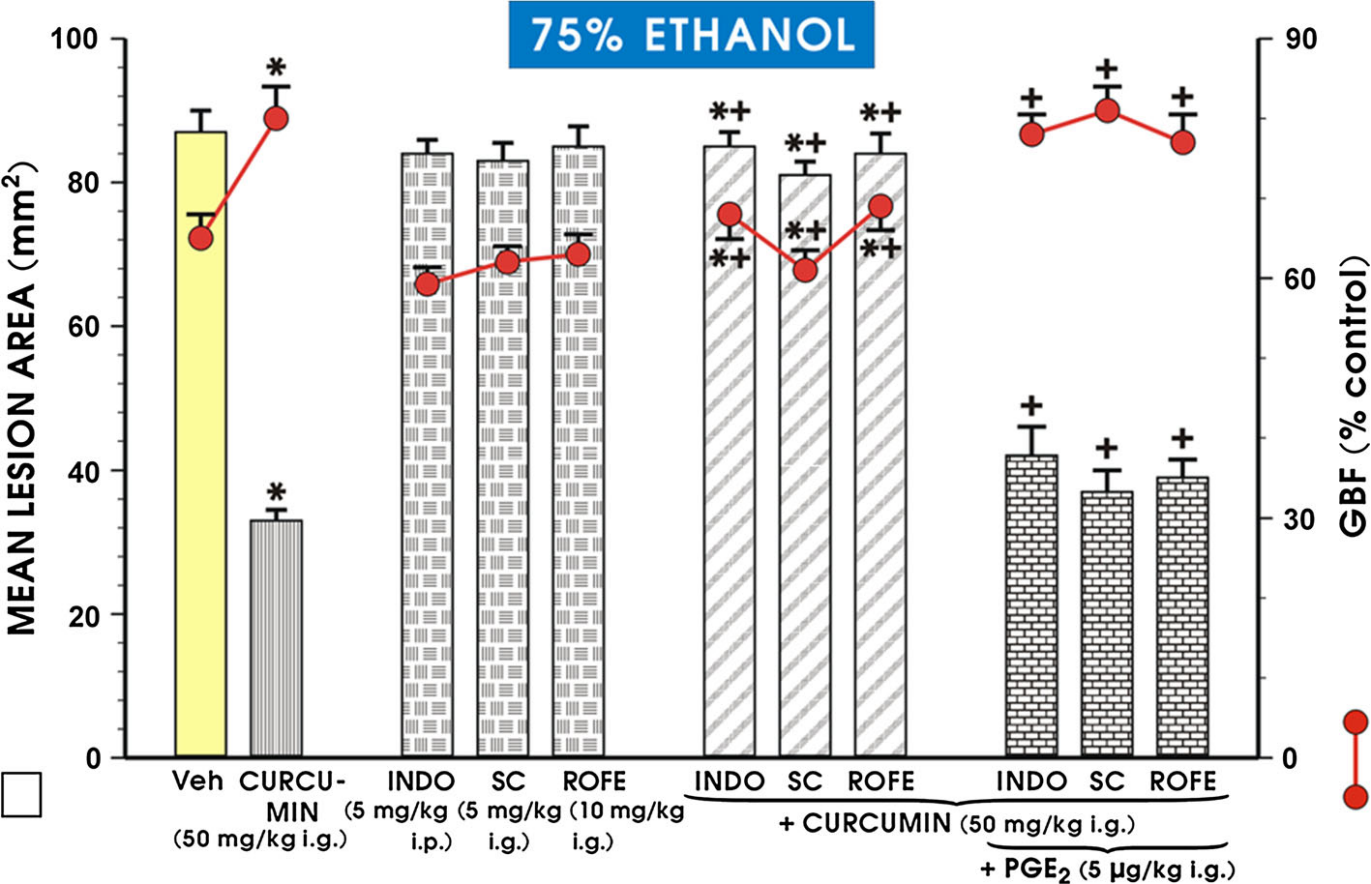
The mean area of ethanol-induced gastric lesions and the alterations in the gastric blood flow (GBF) in rats pretreated intragastrically (i.g.) with curcumin in a dose of 50 mg/kg with or without the concurrent treatment with indomethacin (INDO; 5 mg/kg i.p.), SC-560 (SC; 5 mg/kg i.g.) or rofecoxib (ROFE; 10 mg/kg i.g.) with or without combination with 16,16 dimethyl PGE_2_ (5 μg/kg i.g). Results are mean ± SEM from 6 to 8 rats per group. An *asterisk* indicates a statistically significant (*p* < 0.05) change relative to the vehicle control values. The *asterisk* and *cross* indicate a statistically significant change (*p* < 0.05) relative to curcumin administered alone. The *cross* indicates a significant change (*p* < 0.05) relative to each group treated with COX-1 and COX-2 inhibitor alone

As shown in Fig. 2, curcumin administered in a dose of 50 mg/kg caused a decrease in the area of ethanol-induced gastric damage and an increase in GBF similar to those presented in Fig. 1. The administration of rofecoxib, SC-560 or indomethacin alone failed to evoke statistically significant changes in the area of ethanol-induced mucosal damage and GBF in comparison to the vehicle-control animals exposed to 75% ethanol (Fig. 2). However, when curcumin was co-administered with rofecoxib, SC-560 or indomethacin, a statistically significant increase in the area of ethanol damage and a significant reduction in GBF were observed relative to the corresponding values obtained in rats pretreated with curcumin without concomitant treatment with selective or non-selective COX-1 and COX-2 inhibitors (*p* < 0.05; Fig. 2). The concurrent treatment with 16,16 dimethyl PGE_2_ (5 μg/kg i.g.) combined with either the non-selective or selective COX-1 and COX-2 inhibitors restored the decrease in area of these lesions and the rise in GBF caused by curcumin against ethanol damage (Fig. 2).

**Fig. 3:**
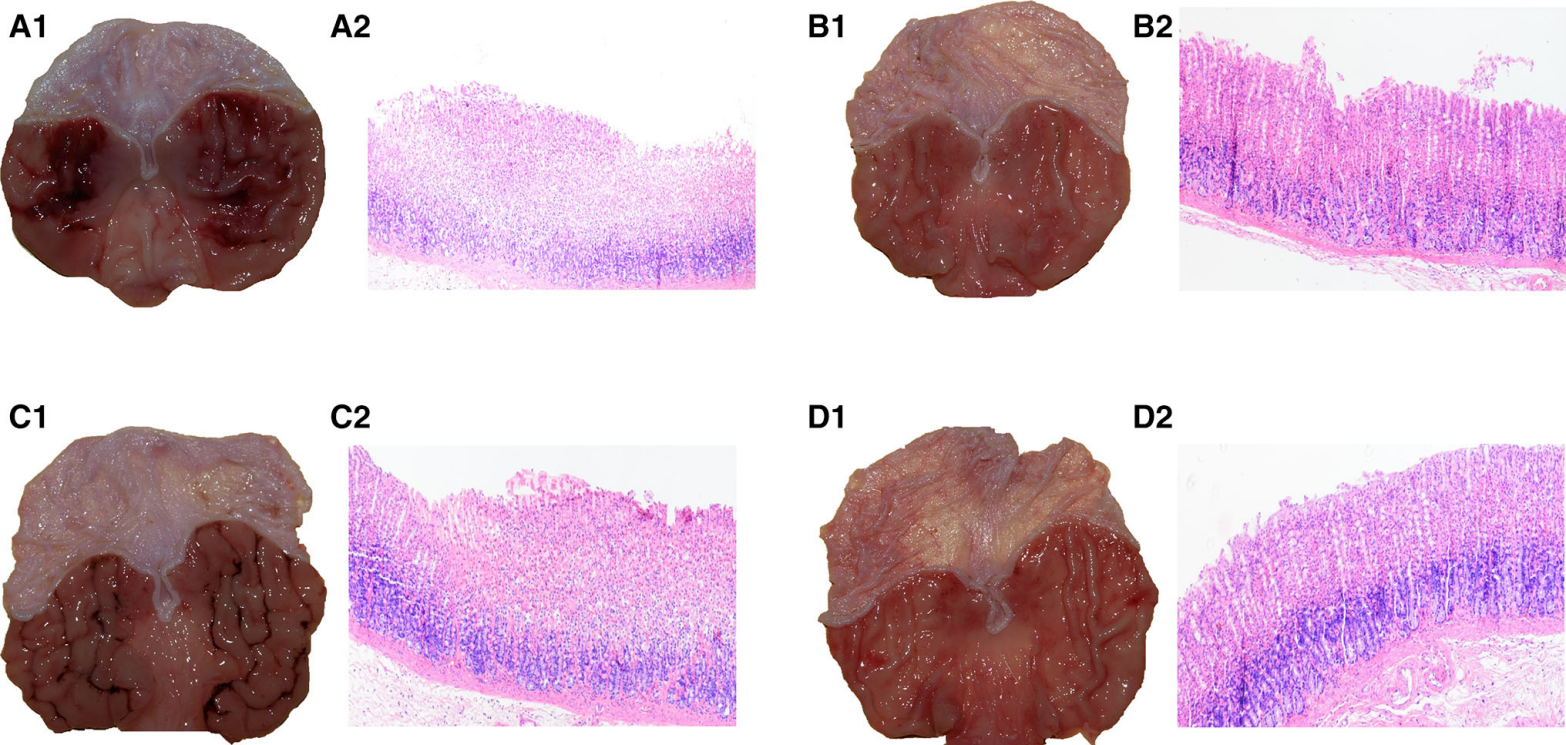
Representative gross macroscopic and microscopic appearance (using H&E staining) of the gastric mucosa pretreated with (**A**) vehicle (saline) or (**B**) curcumin (50 mg/kg i.g.) 30 min before per os instillation of 75% ethanol (1 ml/rat) and curcumin-pretreated gastric mucosa of rats treated with L-NNA (20 mg/kg i.p.) applied alone (**C**) or combined with l-arginine (200 mg/kg i.g.) (**D**) and exposed to 75% ethanol. The severe gastric haemorrhagic lesions are clearly visible in the gastric oxyntic mucosa pretreated with vehicle (saline) and exposed to 75% ethanol (**A1**). The gastric mucosal lesions were markedly reduced in gastric mucosa pretreated with curcumin (50 mg/kg i.g.) and exposed to ethanol (**B1**). In rats pretreated with L-NNA (20 mg/kg i.p.) and administered curcumin (50 mg/kg i.g.), the protective effect of curcumin against ethanol injury was lost (**C1**). When l-arginine (200 mg/kg i.g.) was administered with L-NNA, a curcumin-induced reduction in the formation of haemorrhagic gastric lesions was observed (**D1**). In the vehicle-pretreated rats compromised by ethanol, a severe disruption of gastric mucosa, accompanied by the loss of glandular architecture and leucocyte infiltration, is observed (**A2**). Of note, in curcumin-pretreated gastric mucosa, relatively mild disruption of gastric mucosa along with mild oedema and partial preservation of glandular structure can be observed in comparison with vehicle-control (**B2**). The loss of glandular structure and the presence of deep mucosal lesions were observed in gastric mucosa of rats treated with a combination of L-NNA and curcumin (**C2**). In contrast, the mild disruption of gastric mucosa and a partial restoration of glandular structure along with mild signs of inflammation were observed in gastric mucosa of l-arginine-treated rats administered a combination of L-NNA and curcumin and compromised 30 min later by 75% ethanol (**D2**)

Figure 3A1 shows the representative gross appearance of the gastric mucosa of a rat that was pretreated with vehicle (saline) and received i.g. 1 ml of 75% ethanol 30 min later. The massive haemorrhagic band-like lesions are clearly visible (Fig. 3, panel A1). In the gastric mucosa of rats pretreated with curcumin (50 mg/kg i.g.) and exposed to 75% ethanol, the area of haemorrhagic lesions was markedly reduced, reflecting the gastroprotective effect of this compound (Fig. 3, panel B1). In contrast, when the NO-synthase inhibitor L-NNA (20 mg/kg i.p.) was combined with curcumin, the gastroprotective effect of curcumin was reversed, and the area of gastric lesions was increased relative to curcumin applied alone (Fig. 3, panel C1 vs. panel B1). The combined administration of l-arginine (200 mg/kg i.g.) and L-NNA restored the protective effect of curcumin against ethanol damage as reflected by a decrease in area of macroscopically assessed gastric lesions compared with that observed in rats treated with L-NNA and curcumin but without l-arginine administration (Fig. 3, panel D1 vs. panel C1).

Figure 3 A2–D2 shows the microscopic appearance of the gastric mucosa pretreated with vehicle (saline; A2) and curcumin (50 mg/kg i.g.; B2), both applied i.g. 30 min before the i.g. instillation of 75% ethanol (1 ml/rat). The panels C2 and D2 show the effect of L-NNA (20 mg/kg i.p.) in the presence of curcumin (50 mg/kg i.g.) with or without l-arginine (200 mg/kg i.g.), respectively. In the gastric oxyntic mucosa pretreated with vehicle (saline) and exposed to 75% ethanol (A2), severe destruction of the surface epithelium and extensive oedema of the submucosal layer infiltrated with leucocytes are observed, along with haemorrhagic lesions, some of which clearly penetrate the deeper layers (A2). In addition, there is a distortion of surface epithelium and a lack of normal glandular architecture in vehicle-pretreated gastric mucosa exposed to ethanol (A2). The gastric mucosal haemorrhagic lesions were markedly reduced and the glandular structure was partially preserved in gastric mucosa pretreated with curcumin (50 mg/kg i.g.) and exposed 30 min later to ethanol (B2). In contrast, rats treated with L-NNA (20 mg/kg i.p.) and administered curcumin (50 mg/kg i.g.; C2), exhibited a significant loss of glandular architecture, destruction of the surface epithelium and the appearance of necrotic lesions. This microscopic observation indicates that the protective effect of curcumin against ethanol lesions was evidently lost in curcumin-pretreated animals compromised by L-NNA. When l-arginine (200 mg/kg i.g.) was combined with L-NNA, the gastric mucosal haemorrhagic lesions were reduced in curcumin-treated rats, as reflected by the partial preservation of surface epithelium and glandular structure, and the reduction in leukocyte infiltration and submucosal oedema in the gastric mucosa of these animals (D2).

**Fig. 4:**
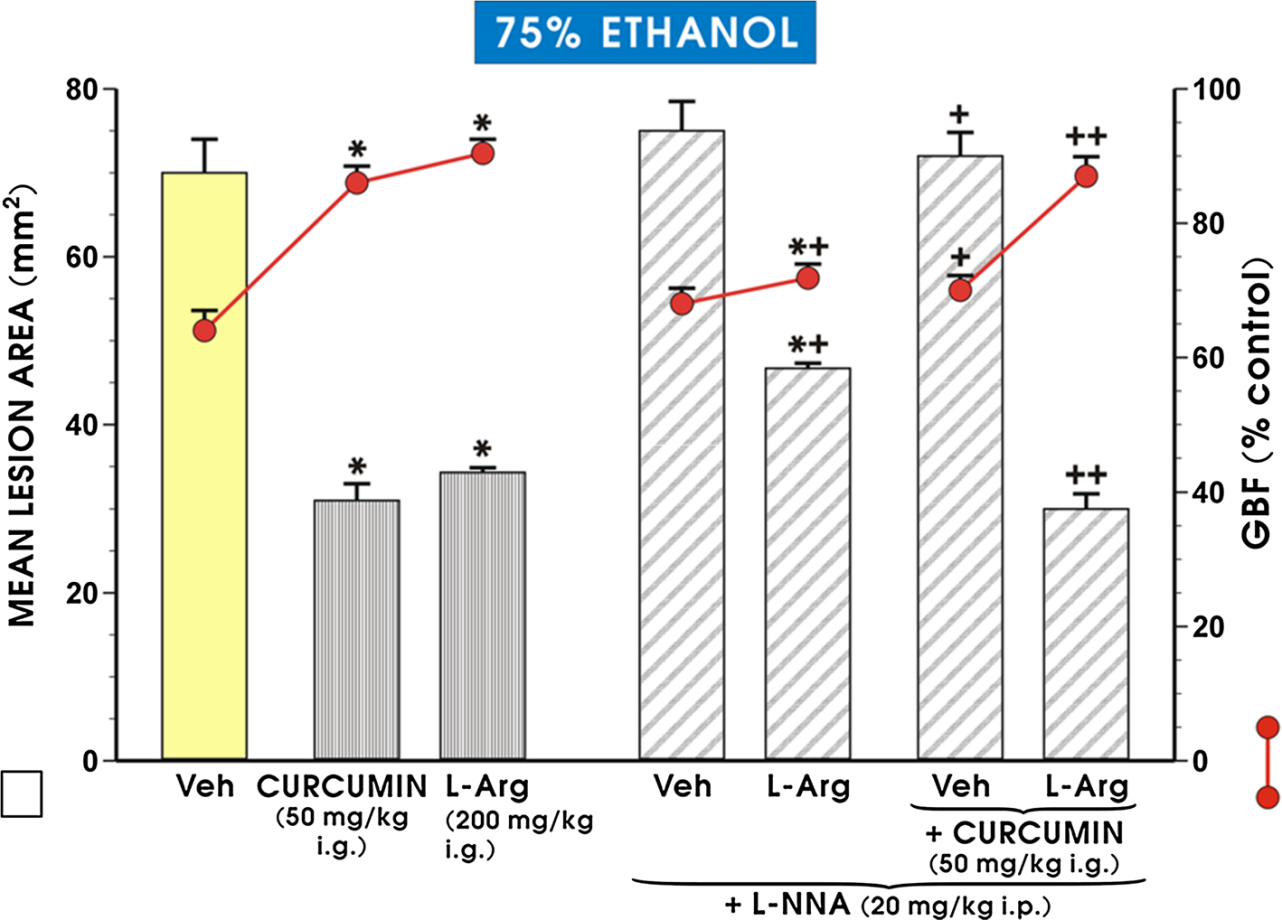
The mean area of ethanol lesions and accompanying changes in the GBF in rats pretreated with curcumin (50 mg/kg i.g.), l-arginine (200 mg/kg i.g.) alone or in those pretreated with a combination of L-NNA administered in a dose of 20 mg/kg i.p. and curcumin (50 mg/kg i.g.) with or without l-arginine (200 mg/kg i.g.). The results are mean ± SEM recorded in seven rats per group. The *asterisk* indicates a significant change (*p* < 0.05) relative to the respective values recorded in the vehicle-control group. The *asterisk* and *cross* indicate a statistically significant change (*p* < 0.05) compared with the values obtained in animals pretreated with l-arg alone. The *cross* indicates a statistically significant change (*p* < 0.05) relative to the values obtained in animals pretreated with curcumin. *Double crosses* indicate a significant change (*p* < 0.05) relative to animals treated with a combination of L-NNA and curcumin

Figure 4 presents the quantitative data derived from experiments with L-NNA (20 mg/kg i.p.) combined with curcumin (50 mg/kg i.g.), with or without l-arginine (200 mg/kg i.g.) administration, on the area of ethanol-induced gastric lesions and the alterations in GBF. The intraperitoneal administration of L-NNA tended to increase, though not significantly, the extent of ethanol-induced gastric mucosal damage and failed to alter the GBF compared to the corresponding values obtained in the vehicle-control group with ethanol alone. However, when L-NNA was combined with curcumin (50 mg/kg i.g.), a statistically significant increase in the area of the ethanol-induced damage and a significant reduction in GBF were observed relative to the corresponding values obtained in curcumin-pretreated rats without L-NNA administration (*p* < 0.05). Pretreating the gastric mucosa of rats with l-arginine (200 mg/kg i.g.) alone prior to exposure to 75% ethanol resulted in a significant reduction in the area of gastric lesions, along with a significant increase in GBF (*p* < 0.05), thereby reaffirming the gastroprotective activity of this amino acid (Fig. 4). The intraperitoneal administration of L-NNA significantly increased the extent of ethanol-induced gastric mucosa damage and significantly decreased GBF compared to the corresponding values obtained in the group with l-arginine alone (*p* < 0.05). The combined administration of l-arginine and L-NNA restored the curcumin-induced decrease in ethanol-derived lesions (*p* < 0.05) and the accompanying increase in GBF (*p* < 0.05) compared to the corresponding values observed in the animals treated only with a combination of L-NNA and curcumin (Fig. 4).

**Fig. 5:**
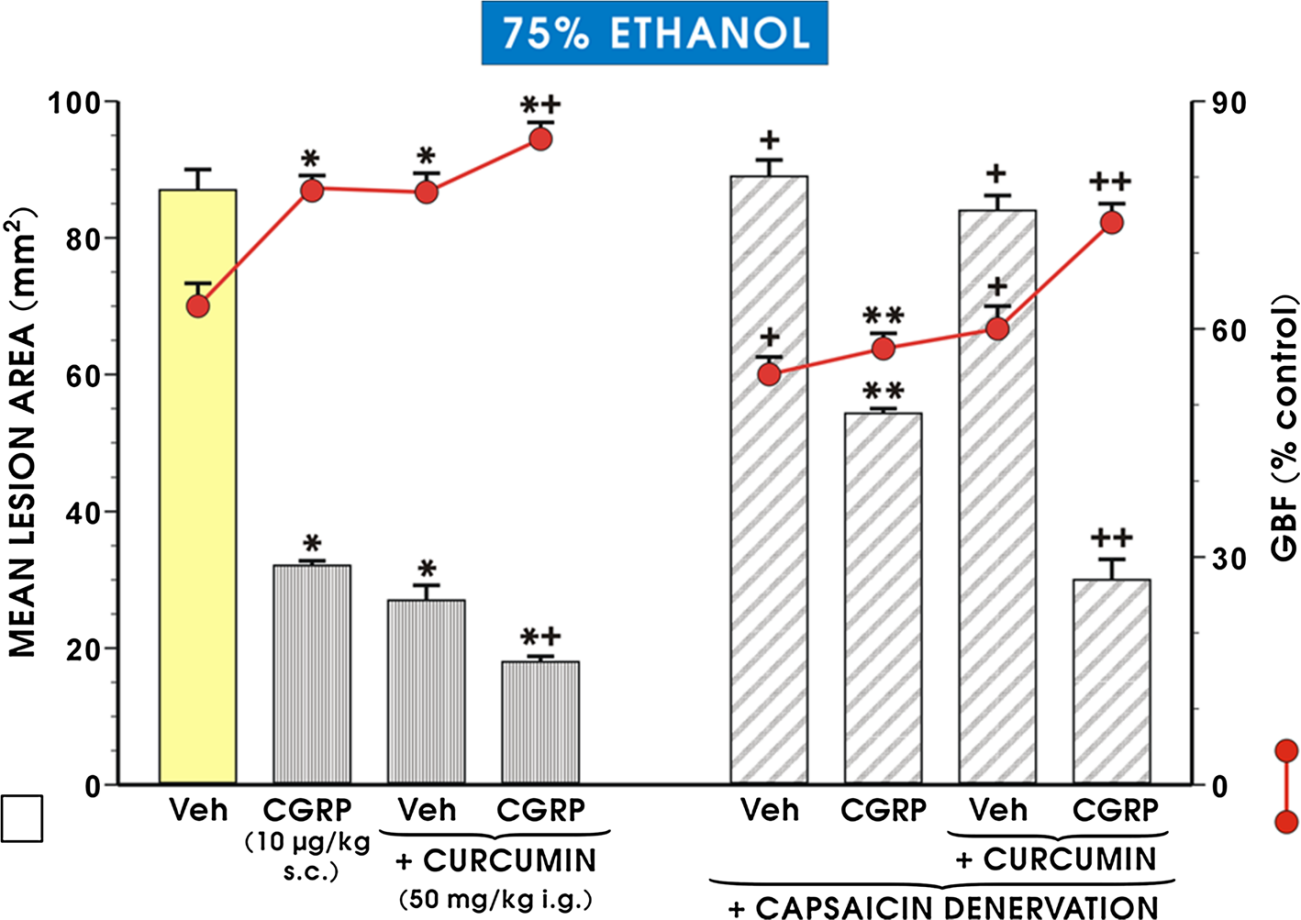
The mean area of ethanol-induced gastric lesions and the changes in gastric blood flow (GBF) in rats with intact sensory nerves or in those with capsaicin denervation pretreated with CGRP (10 μg/kg s.c.) or curcumin (50 mg/kg i.g.) applied alone or administered in combination with CGRP (10 μg/kg s.c.). The results are mean ± SEM from 6 to 8 rats per group. The *asterisk* indicates a significant change (*p* < 0.05) relative to the vehicle-control values. The *double asterisk* indicate a significant change (*p* < 0.05) relative to the values obtained in animals pretreated with CGRP alone. The *asterisk* and *cross* indicate a statistically significant change (*p* < 0.05) as compared to treatment with curcumin alone. The *cross* indicates a significant change (*p* < 0.05) as compared with values obtained in vehicle-control- or curcumin-treated rats with intact sensory nerves. The *double crosses* indicate a significant change (*p* < 0.05) compared with capsaicin-denervated rats treated with curcumin

**Fig. 6:**
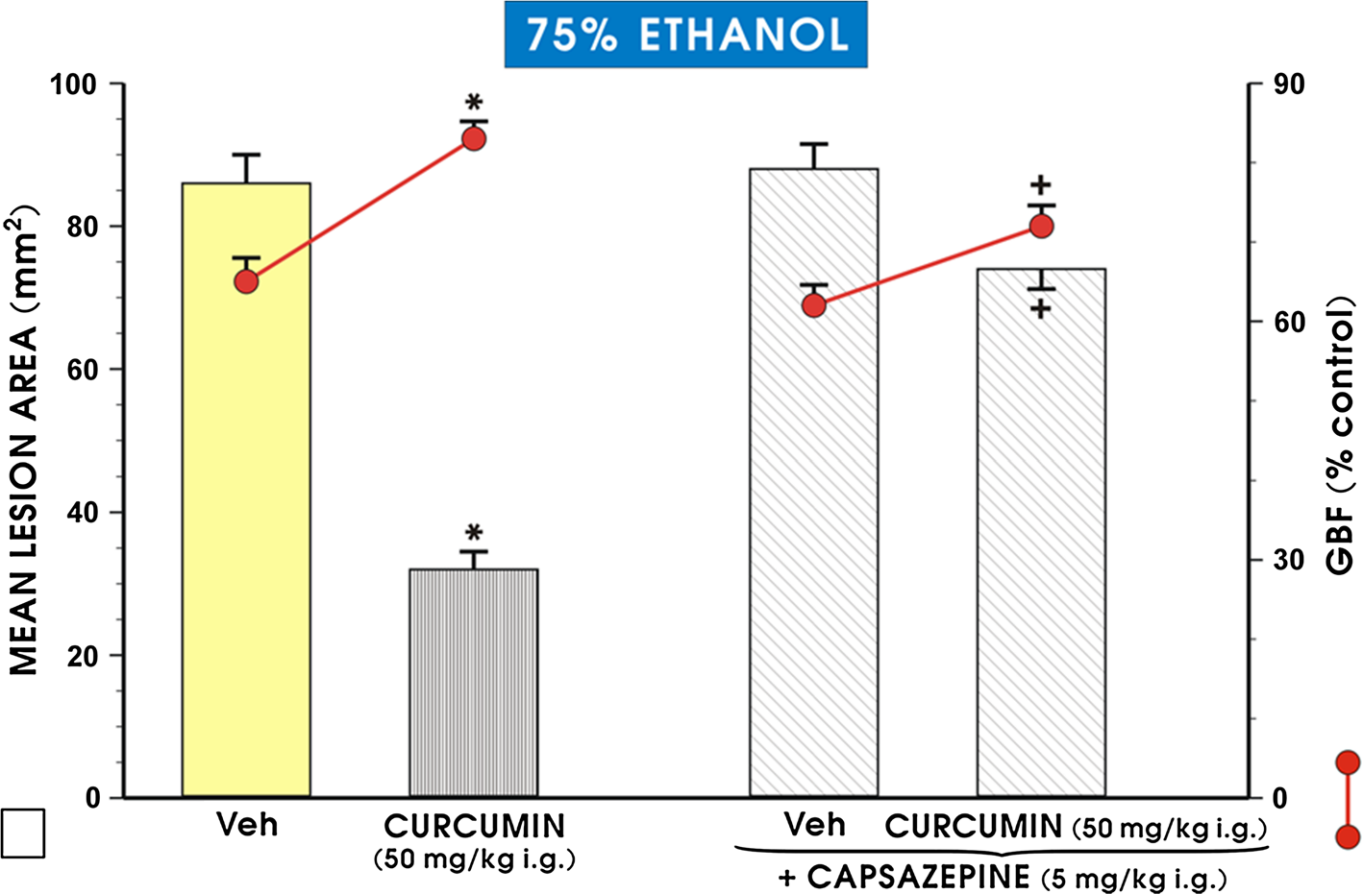
The area of ethanol-induced gastric damage and the changes in gastric blood flow (GBF) in rats pretreated with curcumin (50 mg/kg i.g.), with or without combination with capsazepine (5 mg/kg i.g.). The results are mean ± SEM recorded in seven rats. The *asterisk* indicates a significant change (*p* < 0.05) relative to the vehicle-control values. The *cross* indicates a significant change (*p* < 0.05) relative to animals pretreated with curcumin alone

Figure 5 shows that in rats with intact sensory nerves, pretreatment with curcumin (50 mg/kg i.g.) significantly reduced the area of ethanol-induced lesions (*p* < 0.02) and significantly increased the GBF (*p* < 0.05), similar to the effects presented in Figs. 1 and 2. When CGRP (10 μg/kg s.c.) was applied alone before ethanol, the area of gastric lesions was significantly reduced and the GBF was significantly increased (*p* < 0.05) relative to animals pretreated with vehicle (saline) and exposed 30 min later to ethanol (Fig. 5). The combined administration of CGRP in a dose of 10 µg/kg (s.c.) and curcumin in a standard dose of 50 mg/kg (i.g.) resulted in a further statistically significant reduction in the area of ethanol-induced gastric damage (*p* < 0.05) and an accompanying increase in GBF (*p* < 0.05) as compared with animals pretreated with either curcumin or CGRP alone (Fig. 6). In capsaicin-denervated rats, the i.g. administration of 75% ethanol significantly increased the mean lesion area (*p* < 0.02) and caused a significant fall in the GBF (*p* < 0.02) relative to ethanol-exposed rats with intact sensory nerves (Fig. 5). The curcumin- or CGRP-induced decrease in the area of ethanol damage was reversed in capsaicin-denervated rats, and the GBF was significantly decreased relative to curcumin- or CGRP-pretreated animals with intact sensory nerves exposed to ethanol (*p* < 0.05; Fig. 5). When CGRP in a dose of 10 µg/kg (s.c.) was combined with curcumin (50 mg/kg i.g.) in rats with capsaicin denervation, a significant reduction in the area of ethanol-induced gastric mucosa damage (*p* < 0.05) and a significant increase in GBF (*p* < 0.05) were observed compared with capsaicin-denervated rats treated with curcumin alone and exposed 30 min later to ethanol (Fig. 5).

As shown in Fig. 6, the intragastric application of capsazepine alone did not significantly affect the extent of ethanol-induced damage and had no significant effect on GBF compared to the corresponding values observed in the vehicle-pretreated control animals. The administration of capsazepine combined with curcumin significantly increased the area of gastric lesions (*p* < 0.05) and significantly decreased the GBF (*p* < 0.05) as compared with those obtained in curcumin-pretreated rats without capsazepine administration (Fig. 6).

**Fig. 7:**
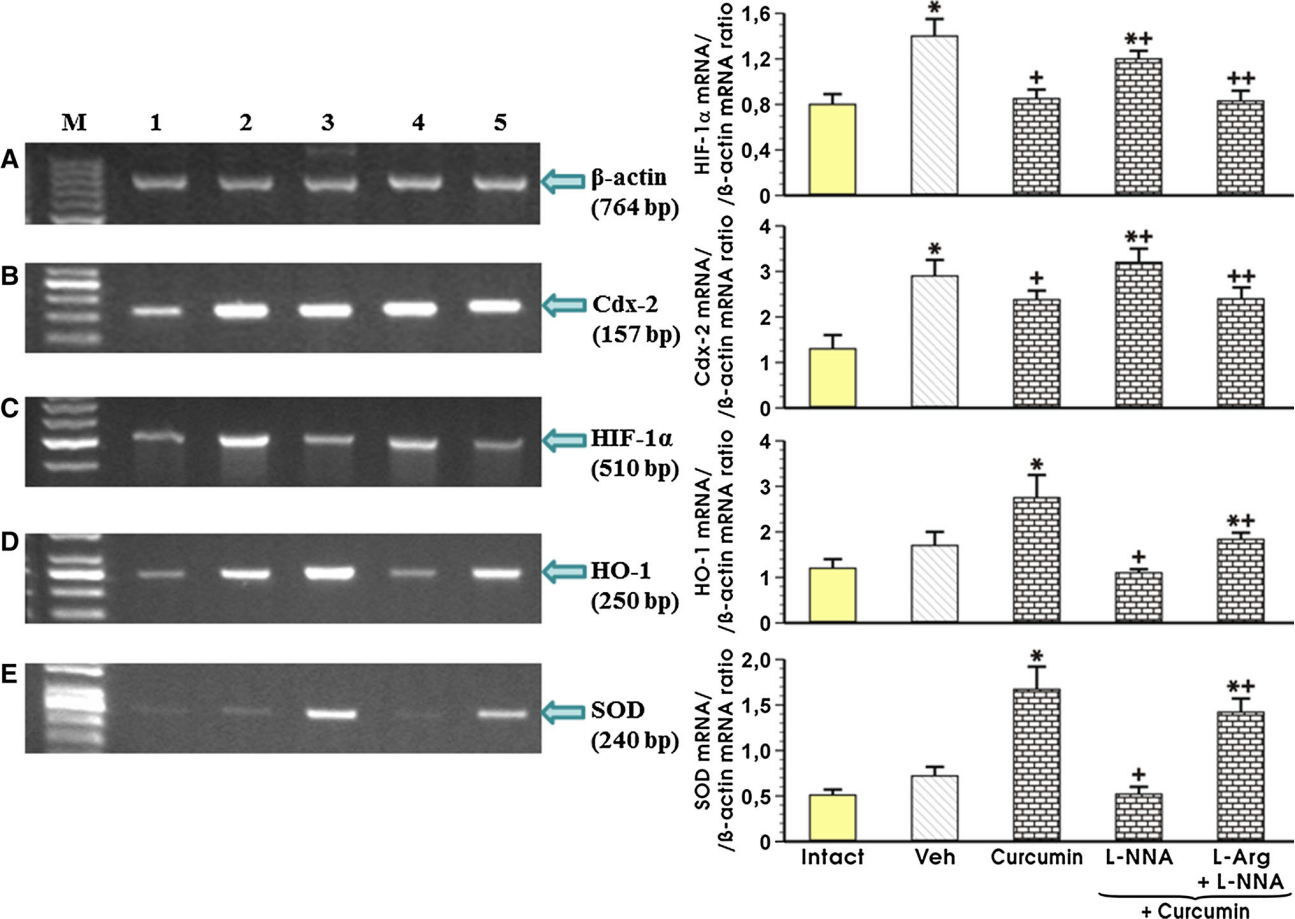
Densitometry analysis of mRNA expression for Cdx-2, HIF-1α, HO-1 and SOD 2 compared to the expression of mRNA for β-actin in the intact gastric mucosa (*line 1*), that with vehicle (saline) and curcumin (50 mg/kg i.g.) pretreatment (*lines 2* and *3*, respectively), and in those administered L-NNA (20 mg/kg i.p.), with or without l-arginine (200 mg/kg i.g.) followed by curcumin (50 mg/kg i.g.) (*lines 5* and *6*, respectively) and exposed to 75% ethanol. Results are mean ± SEM of three experiments. The *asterisk* indicates a significant change (*p* < 0.05) relative to the intact values (graphs 1 and 2) or vehicle-control (graphs 3 and 4). The *cross* indicates a significant change (*p* < 0.05) relative to animals pretreated with the vehicle-control (graphs 1 and 2) or with curcumin (50 mg/kg i.g.) (graphs 3 and 4). The *asterisk* and *cross* indicate a significant change (*p* < 0.05) relative to the values obtained in animals pretreated with curcumin (50 mg/kg i.g.) alone (graphs 1 and 2) or in combination with L-NNA (20 mg/kg i.p.; graphs 3 and 4). *Double crosses* indicate a significant change (*p* < 0.05) relative to those obtained in animals treated with a combination of L-NNA and curcumin (50 mg/kg i.g.)

Figure 7 shows the signal expression of mRNA for HIF-1α, Cdx-2, HO-1 and SOD 2 in the gastric mucosa of intact rats and those pretreated with curcumin (50 mg/kg i.g.), with or without L-NNA alone or in combination with l-arginine. In intact rats, the signal expression of mRNA for HIF-1α, Cdx-2, HO-1 and SOD 2 was negligible. The i.g. application of 1 ml of 75% ethanol in the vehicle-control rats resulted in a statistically significant increase in the gastric mucosal expression of mRNAs for HIF-1α and Cdx-2 and significantly decreased the ratio of HO-1 and SOD 2 mRNA over β-actin mRNA expression compared with intact gastric mucosa (*p* < 0.05; Fig. 7). In curcumin-pretreated rats, the signal of mucosal expression for all of these factors was weak (Fig. 7). The administration of L-NNA significantly increased the expression of mRNA for Cdx-2 and HIF-1α and decreased the expression of mRNA for HO-1 and SOD 2 relative to vehicle-control gastric mucosa exposed 30 min later to 75% ethanol (*p* < 0.05). A semi-quantitative densitometry examination confirmed a statistically significant reduction in the ratio of mRNAs for Cdx-2 and HIF-1α over β-actin mRNA and a significant increase in the expression of HO-1 and SOD 2 mRNAs in curcumin-pretreated animals relative to the vehicle-pretreated control group (*p* < 0.05). The signal expression for HIF-1α and Cdx-2 mRNAs was significantly increased in rats co-administered L-NNA and curcumin (*p* < 0.05; Fig. 7). The semi-quantitative ratio of HIF-1α- and Cdx-2 mRNA confirmed that the concurrent treatment with L-NNA and curcumin significantly increased the expression of HIF-1α- and Cdx-2 mRNA over that recorded in animals treated with curcumin alone (*p* < 0.05). This treatment with L-NNA significantly decreased the HO-1 and SOD 2 mRNA expression in gastric mucosa of curcumin-pretreated rats subsequently treated with ethanol (*p* < 0.05). When l-arginine was co-administered with L-NNA in curcumin-treated rats, the gastric mucosal expression of HIF-1α and Cdx-2 mRNA was significantly decreased compared with that detected in rats that were not administered l-arginine (*p* < 0.05; Fig. 7). In contrast, the concurrent treatment with l-arginine and L-NNA significantly increased the expression of HO-1 and SOD 2 mRNAs as compared with that observed in that without l-arginine administration (*p* < 0.05; Fig. 7).

## Discussion

The present study was designed to determine the gastroprotective mechanism of curcumin, a product of curcuma longa [1–4, 15, 16], against the formation of acute gastric mucosal lesions induced by ethanol. We confirmed that ethanol, a topical irritant which acts through direct contact with the gastric mucosa, caused widespread haemorrhagic lesions in the rat gastric mucosa. Similar haemorrhagic ethanol-induced gastric mucosal lesions have been reported in the human stomach [33]. The preservation of the integrity of gastric mucosa exposed to a variety of damaging agents is dependent on the balance between the epithelial and subepithelial components regulated by the proliferative zone of gastric glands and the mucosal defence mechanisms such as undisturbed mucosal blood flow, mucus and alkaline secretion and the restitution and proliferation of mucosal cells [34, 35]. The endogenous prostaglandins, sulfhydryl compounds, the activation of endothelial and mucosal generation of NO, hydrogen sulfide (H_2_S) and carbon monoxide (CO) contribute to mucosal integrity and gastroprotection [36–38].

Here, we demonstrated that curcumin, when given intragastrically, afforded dose-dependent gastroprotection against ethanol damage, while increasing GBF and plasma gastrin levels. A previous study reported that treatment with Zn(II)-curcumin increased proliferative activity and enhanced antioxidant mechanisms of gastric mucosa injured by necrotizing agents [16]. In another work, curcumin reduced oxidative stress and acute gastric mucosal damage by preventing a reduction in the activity of the enzymes glutathione dehydrogenase (GSH) and SOD known to scavenge reactive oxygen metabolites [39]. The reduced expression of proinflammatory mediators, the generation of free nitrogen radicals, and the inhibition of apoptosis and increase in cell proliferation in the gastric mucosa have been implicated in the mechanism of curcumin-induced gastroprotection against a variety of non-topical and topical ulcerogenes [40, 41]. Following an increase in the dose of curcumin, we observed a concomitant increase in the GBF and plasma gastrin levels, suggesting that curcumin protection could involve an improvement in gastric microcirculation possibly mediated by gastrin, which is known to exert a trophic effect on gastric mucosa [42, 43]. Moreover, gastrin has been shown to intensify cellular metabolism, increase GBF and the proliferative activity of cells, and enhance the repair process and mucosal healing by strengthening the restitution and regeneration of surface epithelial cells [44]. Gastrin has been found to exhibit potent gastroprotective and hyperemic activity against injury evoked by necrotizing irritants such as ethanol, in part dependent on endogenous PG and NO [45]. Therefore, we speculate that the observed increase in plasma circulating gastrin may help to explain not only the protective but also the hyperemic activity of curcumin in the stomach injured by ethanol.

The novelty of our work is that it suggests that curcumin-induced protection may be dependent upon the reduced mRNA expression of pro-inflammatory mediators HIF-1α and Cdx-2 in the gastric mucosa. Indeed, the administration of 75% ethanol in vehicle-control rats increased the expression of mRNA for HIF-1α and Cdx-2 in the gastric mucosa, but this effect was abrogated by curcumin. Moreover, curcumin increased the mRNA expression for anti-inflammatory markers HO-1 and SOD 2 downregulated by ethanol, which seems to explain the beneficial role of this compound in gastroprotection. Therefore, we speculate that the activation of antioxidant enzymes HO-1 and SOD 2 can contribute to curcumin-induced protection. Our results regarding curcumin-controlled expression of antioxidant and anti-inflammatory enzyme HO-1 are in accord with a previous report that curcumin afforded protection against H_2_O_2_-mediated apoptosis via an HO-1-dependent mechanism [46].

The gastroprotective effect of curcumin may result from its inhibition of prostaglandins derived from COX-2, but this compound has no direct or indirect inhibitory effect on COX-1 expression and activity in various systems [47, 48]. PG considered as the major products of COX-1 and COX-2 activity, are regarded as classic mediators of cytoprotection and when applied exogenously in the non-antisecretory doses, they are able to prevent the mucosal damage induced by necrotizing substances (e.g. absolute ethanol) [47, 48] and contribute to mechanisms of gastroprotection, gastric adaptation to damaging agents and the healing of acute and chronic ulcerations [49–51].

In our study, the non-selective (indomethacin) and selective COX-1 (SC-560) and selective COX-2 (rofecoxib) inhibitors, which were used in doses that significantly reduced the gastric mucosal generation of PG [52, 53], were not sufficient in themselves to exacerbate the ethanol-induced gastric mucosal injury. However, when indomethacin, rofecoxib and SC-560 were combined with curcumin, a significant increase was observed in the area of ethanol damage, accompanied by a decrease in GBF, compared with animals pretreated with curcumin alone. Concurrent treatment with a synthetic analogue of PGE_2_ combined with these COX-1 and COX-2 inhibitors restored the protective and hyperemic effects of curcumin against ethanol damage. This indicates that PG synthesized by both COX isoforms, namely, COX-1 and COX-2 can mediate the gastroprotective effect of curcumin.

NO acts an important mediator of gastroprotection and ulcer healing [52, 54]. We observed that the blockade of NO synthesis in the gastric mucosa following the application of L-NNA suppressed the gastroprotective effects of curcumin, and this effect was accompanied by a marked reduction in the GBF. Moreover, the application of l-arginine in a dose which attenuated ethanol-induced gastric lesions, together with L-NNA, restored the protective and hyperemic activity of curcumin against ethanol injury. The L-NNA reversed the curcumin-induced decrease in the expression of HIF-1α and Cdx-2 mRNAs. In line with this observation, the concurrent treatment with l-arginine, a substrate for NO synthase combined with L-NNA attenuated an increase in the expression of HIF-1α and Cdx-2 caused by L-NNA. This indicates that NO could play an important role in the protective activity of curcumin due to its hyperemic and anti-inflammatory properties.

In our present study, exogenous CGRP, the major sensory nerve mediator [44], attenuated gastric lesions and significantly raised the GBF. In contrast, the capsaicin denervation augmented the extent of ethanol-damage and decreased GBF relative to the group of rats with intact sensory nerves. The capsaicin denervation eliminated the protective activity of curcumin, and to some extent that of CGRP applied alone, and reduced the accompanying GBF. However, when CGRP was combined with curcumin in capsaicin-denervated rats, a partial restoration of the gastroprotective and hyperaemic effects of curcumin was observed. To the best of our knowledge, this constitutes the first demonstration that the sensory afferent fibres releasing neurotransmitters such as CGRP together with NO, possibly derived from cNOS activity, could be involved in the mechanism of curcumin-induced gastroprotection against ethanol injury. Taken together, this leads us to speculate that the mechanism of curcumin-induced protection could involve an increase in the activity of the afferent sensory nerves and release of vasoactive CGRP, thus causing vasodilation and an increase in GBF.

Recent studies have demonstrated the co-localization of CGRP with the vanilloid receptor (TRPV-1) in the gastric mucosa of rodents acting as target, along with other neurotransmitters released from sensory fibres [55, 56]. In our present study, the administration of capsazepine, the TRPV1 receptor antagonist, which did not further intensify the ethanol-induced damage of gastric mucosa, substantially attenuated the gastroprotective activity of curcumin. Thus, based on experiments with capsazepine, we speculate that in addition to CGRP and NO, TRPV1 receptors can participate in the gastroprotective mechanism of curcumin. The possibility that curcumin can act directly through the activation of the TRPV1 receptors cannot be excluded, because the vanilloid structure of curcumin has been implicated in activation of the TRPV1 receptor, resulting in enhanced apoptosis [57].

In summary, our study demonstrates that curcumin affords dose-dependent gastroprotective effects against ethanol damage, accompanied by hyperemia and an increase in plasma gastrin levels. On one hand, the intragastric administration of curcumin affords protection against ethanol lesions via an increase in gastric microcirculation and the inhibition of expression of proinflammatory markers HIF-1α and Cdx-2 in gastric mucosa exposed to ethanol. On the other hand, curcumin increased the expression of mRNA for anti-inflammatory factors HO-1 and SOD 2 in the gastric mucosa injured by ethanol. Endogenous PG and NO, as well as the activity of capsaicin-sensitive visceral sensory fibres releasing CGRP and the vanilloid receptor (TRPV-1), appear to contribute to the mechanisms underlying the gastroprotective and hyperemic effects of curcumin.

## Electronic supplementary material

Below is the link to the electronic supplementary material.

Supplementary material 1 (DOCX 14 kb)
