# Supplementary material for: Mechanisms of curcumin-induced gastroprotection against ethanol-induced gastric mucosal lesions
Source: J Gastroenterol. 2017 Aug 30;53(5):618–30. doi: 10.1007/s00535-017-1385-3 (PMC5910495; doi:10.1007/s00535-017-1385-3)
Supplement: Supplementary file 1 — Supplementary material 1 (DOCX 14 kb) [file 535_2017_1385_MOESM1_ESM.docx]

Tab. S1. The sequence of the primers, annealing temperature and the size of the PCR product in the reverse transcription reaction determining the expression of the genes of Cdx-2, Hif1-α, HO-1 and SOD 2.

| **Gene** | Primer sequence | Size of the PCR product |
| --- | --- | --- |
| **β-actin** | Forward: 5’-TTG TAA CCA ACT GGG ACG ATA TGG-3’,  Reverse: 5’-GAT CTT GAT CTT CAT GGT GCT AGG-3’ | 764 bp |
| **Cdx-2** | Forward: 5’-CGC GAG GAC TGG AAT GGC TA-3’ Reverse: 5’ - CCG GAT GGT GAG GGT GAT GA-3’ | 157 bp |
| **Hif1-α** | Forward: 5’-TCT GGA CTC TCG CCT CTG-3’G-3  Reverse: 5’-GCT GCC CTT CTG ACT CT- 3’ | 510 bp |
| **HO-1** | Forward: 5’- CTT GCA GAG AGA AGG CTA CAT GA-3’ Reverse: 5’- AGA GTC CCT CAC AGA CAG AGT  TT -3’ | 250 bp |
| **SOD 2** | Forward: 5’- CAG CCT TGT GTA TTG TCT TC-3’  Reverse: 5’- GCT TCT CTC GTC TCC TTG CT-3’ | 201bp |
